# Healthcare Digitalisation and the Changing Nature of Work and Society

**DOI:** 10.3390/healthcare9081007

**Published:** 2021-08-06

**Authors:** Henrik Skaug Sætra, Eduard Fosch-Villaronga

**Affiliations:** 1 Faculty of Computer Sciences, Engineering and Economics, Østfold University College, N-1757 Halden, Norway; 2 eLaw Center for Law and Digital Technologies, School of Law, Leiden University, 2311 EZ Leiden, The Netherlands; e.fosch.villaronga@law.leidenuniv.nl

**Keywords:** healthcare, digitalisation, artificial intelligence, robotisation, work

## Abstract

Digital technologies have profound effects on all areas of modern life, including the workplace. Certain forms of digitalisation entail simply exchanging digital files for paper, while more complex instances involve machines performing a wide variety of tasks on behalf of humans. While some are wary of the displacement of humans that occurs when, for example, robots perform tasks previously performed by humans, others argue that robots only perform the tasks that robots should have carried out in the very first place and never by humans. Understanding the impacts of digitalisation in the workplace requires an understanding of the effects of digital technology on the tasks we perform, and these effects are often not foreseeable. In this article, the changing nature of work in the health care sector is used as a case to analyse such change and its implications on three levels: the societal (macro), organisational (meso), and individual level (micro). Analysing these transformations by using a layered approach is helpful for understanding the actual magnitude of the changes that are occurring and creates the foundation for an informed regulatory and societal response. We argue that, while artificial intelligence, big data, and robotics are revolutionary technologies, most of the changes we see involve technological substitution and not infrastructural change. Even though this undermines the assumption that these new technologies constitute a fourth industrial revolution, their effects on the micro and meso level still require both political awareness and proportional regulatory responses.

## 1. Introduction

Digital technologies have profound effects on all areas of modern life, including life at the workplace. By digitalisation we here refer to all forms of digital technologies, including artificial intelligence (AI) and robot technologies. Certain forms of digitalisation entail simply exchanging digital files for paper, while more complex instances involve computers and machines that perform a wide variety of tasks. From self-service checkouts in major supermarkets, IKEA, and the post office, to automated warehouses, it is becoming more and more evident that robots are not merely performing tasks, but taking over jobs from humans [1,2,3]. While some are wary of the displacement of humans that occur when robots perform tasks previously performed by humans, others argue that robots only perform the tasks that should have been carried out by robots in the very first place, and never by humans [4]. Some have even argued that robots should be designed and conceived of as some form of slaves [5], although this can have repercussions for human relationships [6].

Researchers and consultants have examined how jobs are increasingly susceptible to automation for some time now. For example, studies claim that big data techniques could substitute non-routine cognitive tasks and that increased robot dexterity will allow robots to perform an increasing number of manual tasks previously thought to require humans [7,8,9]. A recent and extensive quantitative study on industrial robots and human replacement also shows that, although not in alarming numbers, there is a tendency towards worker replacement in industrial environments due to the productivity robots offer [10]. The World Economic Forum [11] suggests that, instead of replacing existing occupations and job categories, robots and artificial intelligence (AI) will substitute specific tasks to free workers up to focus on new tasks. This notion is taken further by Danaher [3], who argues that work in general is, in fact, something that most people would benefit from being freed from. Along the same lines, the European Parliament points out that healthcare robots may ease the work of care assistants by performing automated tasks [12]. This technology will allow caregivers to devote more time to diagnosis and better-planned treatment options.

Understanding the effects of digitalisation in work life requires us to understand the effects of digital technology on the tasks we perform. Usually, these effects entail further impacts that are not always foreseeable, thus a broader and more comprehensive analysis is required. In this article, the changing nature of work in the health care sector is used as a case to analyse such change on three levels: the societal level (macro), the organisational level (meso), and the individual level (micro). The societal level involves asking whether the new technologies in question entail technological change of a substitutional or infrastructural kind [13]. For example, some argue that digital technologies are now taking us towards a fourth industrial revolution (4IE from hereon) [14], and this would entail that technologies, such as artificial intelligence (AI), big data, and modern robotics, lead to change in the technological infrastructure. In addition, we argue that it is important to simultaneously ask how these changes—of both kinds—affect the experience of work from the organisational and individual perspectives. We, here, use the theories of Kaptelinin [15] and Norman [16] to examine the changing nature of tasks on an individual level, while they also allow us to examine change on an intermediate (meso) level.

Our contribution in the perspective article explores how distinguishing between the micro, meso, and macro level and changes to activities view from the system and personal level allows us to better understand the nature of digitalisation and the technological change it entails for the healthcare sector. While the changes in the healthcare sector involve particular technologies and occupations, we argue that these examples and the analytical framework we develop based on these are relevant to understanding other sectors as well, and also technological change in general. We emphasise the importance of a layered analytical approach, which precludes us from going into detail on all the particular technologies and occupations in the sector, but allows us to understand the actual magnitude of these changes and foster informed regulatory and societal responses. By analysing these transformations following a layered micro-meso-macro approach, we can encourage an informed and proportionate response, help preserve the rule of law, and avoid what has been called “regulatory madness” (in French *la folie normative*) [17]. Such a madness implies a rushed over-regulatory response that will not necessarily solve the problems it aims to address, while it could hamper innovation without providing a usable compass to guide society and technological development. On the contrary, this could contribute to the creation of “legal bubbles” that arise in times of innovation and increased economic investment in areas in which legislation is still immature, whereby consequences of the new technologies in question are still partly unknown [18]. A layered approach would help prevent the disruptive consequences of ignoring the implications of technological development at different levels.

We argue that, while AI, big data, and robotics are revolutionary technologies, most of the changes we see involve technological substitution, and not infrastructural change. This entails that the notion of a fourth industrial revolution might not be the most useful concept for understanding the changes we now see happening through digitalisation in the healthcare sector. Such changes might certainly affect the experience of work life for a large number of individuals, but this does not necessarily lead to the conclusion that the technological structure is changing in radical and new or revolutionary ways. Not having a clear idea about the nature and extent of these transformations may lead to regulatory, economic, political, and societal responses that are disproportionate to the nature of these changes and, therefore, may have a range of unintended effects.

This article is structured as follows. In Section 2 we present a selection of key examples of digitalisation and automation in the healthcare sector. In Section 3, we present a theoretical framework for analysing these examples, and we apply this framework to the examples in the discussion in Section 4. More focused studies, based, for example, on systematic literature reviews of the kinds of changes we here describe within a particular part of the healthcare sector, will be a natural next step for testing the approach and hypotheses put forth in this article.

## 2. Healthcare Automation Transformations

Healthcare automation based on digital technologies are here examined through two major technologies: AI and robotics. AI is expanding the frontiers of medical practice [19,20]. The increased availability of data, improved computing power, and advances in machine learning has led to this proliferation of AI systems [21,22]. Various medical domains previously reserved for human experts are increasingly augmented or changed thanks to the implementation of AI, including decision making in clinical practice (e.g., disease diagnosis, automated surgery, patient monitoring, foetus monitoring in the prenatal phase), translational medical research (such as improvements in drug discovery, drug repurposing, genetic variant annotation), and tasks related to basic biomedical research (e.g., automated data collection, gene function annotation, and literature mining) [23,24,25]. In addition to automation of data collection and improved data from traditional sources, researchers have also found new sources of data that are used in healthcare research, such as data from social networks, for example Twitter [26].

One example of a research field that is highly relevant for both AI and robotics in the healthcare sector is research related to dementia, which is expected to be a key challenge for the healthcare sector—and society in general—in the years to come [27,28]. A recent study shows that a deep learning model could predict Alzheimer on average six years before the final diagnosis was made [29]. In another recent study from a related field, researchers show that an AI-powered triage and diagnostic system produces differential diagnoses with an accuracy comparable to human doctors in precision and recall [30]. Although these systems only outperform human doctors in certain cases, their findings show that, on average, the AI system assigned triages more safely than human doctors.

A study from the University of North Carolina School of Medicine tested IBM Watson for Genomics against 1018 cancer diagnoses targeting tumour and normal tissue DNA sequencing [31]. The results showed that human oncologists agreed with 99% treatment plans from IBM Watson. Moreover, Watson ascertained treatment options human doctors missed in 30% of the cases. In a different study, Watson analysed 638 treatment recommendations for breast cancer with a human-Watson concordance rate of 93% [32]. These technologies may have the potential to predict healthcare-related outcomes, including genetic disorders or suicide risk, leading to an earlier intervention, and potentially save more lives [33,34].

A key challenge associated with the use of AI systems in the healthcare sector is the lack of transparency and explainability [21], a topic which is receiving increasing attention from regulators, as seen, for example, in the European General Data Protection Regulation (GDPR) [35]. As the healthcare sector is what must be considered a security-and privacy-sensitive domain, transparency and the development of means to uncover, for example, bias in decision-making systems, is vital [21]. Such issues highlight the need for a suitable regulatory approach and response, which applies both to AI and robotics systems. 

Although decision support systems that combine aggregated patient information have existed for a while, progress in this domain conveys the impression that machines for specific tasks will soon outperform humans. The fear is that, even in the healthcare sector, which was previously portrayed as relatively immune to automation, there is a clear tendency for professional tasks to become increasingly susceptible to digitalisation or automation. Routine tasks—both cognitive and physical—are already being automated on a large scale. However, big data techniques also enable us to substitute humans for non-routine cognitive tasks, and progress in robot dexterity could allow robots to perform increasingly complex manual tasks and hence lead to what is perceived as a profound transformation of healthcare workplaces. However, how profound and fundamental are these changes really? That is the question we return to in Section 4.

We now turn to the other key technology being examined in the article, namely robots. One example we will consider in Section 4 is the introduction of robots in care, with a particular focus on care for the elderly [28,36]. In addition to social robots, who mainly allow for the automation of therapeutic and welfare increasing interventions, there are a large number of robots that provide physical assistance while also functioning as an interface to various digital technologies [37]. Assistive technology has been developed in order to, for example, help with feeding, lifting, and washing [38]. There are also a number of ways in which such technologies can be used to sense, monitor, and alert when particular situations occur, such as an elderly person falling in their bathroom [39]. These kinds of technologies might be applied in eldercare facilities, but they will also allow an increasing number of people to age at home [40].

Nevertheless, robots are not only used in a care setting. Robot-assisted surgery (RAS) is associated with a number of benefits that we will return to shortly, and introducing a robot to the doctor-patient relationship changes how surgeries are performed. RAS extends the abilities of the doctor, but it also presents new challenges. A revision of 14 years of data from the Food and Drug Administration (FDA) shows that robot surgeons can cause injury or death if they spontaneously power down mid-operation due to system errors or imaging problems [41]. Broken or burnt robot pieces can fall into the patient, electric sparks may burn human tissue, and instruments may operate unintendedly; all of which may cause harm, including death [41]. Moreover, as surgical robots’ perception, decision-making power and capacity to perform tasks autonomously will increase, and the surgeon’s duties and oversight over the surgical procedure will inevitably change. Other issues relating to cybersecurity and privacy will also become more significant [42].

Additionally, security vulnerabilities may allow unauthorised users to remotely access, control, and issue commands to robots, potentially causing harm to patients [43]. Despite its widespread adoption for minimally invasive surgery (MIS), a non-negligible number of technical difficulties and complications are still experienced during surgical procedures performed by surgical robots. To prevent or, at least, reduce such preventable incidents in the future, advanced techniques in the design and operation of robotic surgical systems and enhanced mechanisms for adverse event reporting are important [41]. 

While these ethical considerations are crucial for achieving responsible and beneficial digitalisation, we must also note that RAS, for example, provides a wide range of benefits, as surgery might be made more reliable, precise, and effective, and expert surgeons will be available to a broader range of potential patients. Such benefits must be weighed against the potential downsides just discussed, and policy related to such technologies involves examining whether technologies, such as RAS, are overall beneficial for patients, as no technology—and no human—can ever be perfectly safe or error-free. 

The introduction of highly sophisticated machines in the healthcare domain may entail several changes, but the nature of these changes may not be immediately apparent. This is because analyses of the changes caused by the insertion of a particular technology often fail to consider the broader consequences this may have at multiple levels, including the individual, the organisational, and the social. For instance, robot-mediated surgeries may have implications for new roles and responsibilities of medical practitioners and staff (individual), the allocation of responsibility and insurance (organisational), or even the education of future medical doctors (societal) [44]. In the following section we introduce a layered theoretical framework that helps in understanding and differentiating between the various consequences of technology adoption—either positive or negative—at different levels.

## 3. Theoretical Framework

To examine how work in the healthcare sector is changing, we develop a layered framework for analysing these changes at multiple levels: social and economic (macro), intermediate or organisational (meso), and individual (micro) levels. The macro level relates to large- and long-scale impacts on societies and economies as production systems [45], which is the level the discussions of industrial revolutions usually refer to [13]. When the focus is shifted to the intermediate level, including organisations and the relationships between organisations, institutions, political bodies, and regulators, we refer to the intermediate or meso level [45]. Finally, the micro level refers to changes that affect individuals, or that are limited to changes within organisations or groups [45]. With such a broad focus, our main goal is to provide a framework for analysing the effects of digitalisation, and the examples we use cannot provide a complete picture of how healthcare is changing. It will, however, provide a starting point for this discussion, which that can subsequently be tested, supplemented and continued in more focused and empirical research. 

First, we distinguish between substitutional and infrastructural technological change [13]. Technological substitution involves using technology to perform tasks in a more efficient manner *within* the existing sociotechnical framework [13]. If a technology enables a worker to do things more quickly, for example, without really changing the nature of the work, technological substitution allows for increased productivity without broader implications for the socio-technical system. Technologies may also lead to more fundamental changes, however, if they involve changes in the very infrastructure of work. Electrical power and the combustion engine, for example, are examples of technologies that are seen as changing technological infrastructure. Such changes entail changes in the broader socio-technical structure, involving, for example, what sort of tasks people are needed for, the educational requirements for working with the new technologies, structural changes in the companies, whether technologies allow for production, and work in larger or smaller units. This, in turn, may change society itself, by the changes it leads to in income structures, education, and even residential patterns [13]. 

In the case of welfare technology, imagine the effects of social robots in elder care. When a robot seal, for example, Paro, is introduced to the elderly with dementia [36], how does this change the work of the caretakers? Does it make the caretakers more effective, as they have a new tool that enables them to care for more elderly or provide better care for the same number? Or does the introduction of robots change the nature of the work, and consequently entail more fundamental changes for those who work in the care sector, including changes regarding what sort of skills are required, and, not least, how many people work in the sector? The first situation in which work is simply made a bit more effective would be an example of technological substitution, while the latter might entail changes fundamental enough to be infrastructural. 

Sharkey and Sharkey [38], Coeckelbergh [46], and Sparrow [47] reason about situations in which robots have completely replaced humans in elder care. Such dystopian scenarios are not necessarily infrastructural just because of the scope of substitution in care facilities. If robots perform the tasks and actions almost precisely as humans would do, and if this replacement does not entail wider societal effects related to education, employment, the need for substantial relocation for workers, or changed economic structures, for example, the change might still be substitutional [13]. In addition, the use of technology in particularly sensitive domains—such as domains involving care—are traditionally assumed to require a ‘human touch’ and may entail even broader long-term consequences for society [48].

This takes us into the domain of human–computer interaction (HCI), in which the relationship between humans and computers is studied. In this article, we limit this discussion to the introduction of two similar, but slightly different, perspectives on such interaction: the cognitive approach and activity theory [15,16]. Norman [16] is a proponent of the cognitive approach in the field, and he explains that there are two different views of artefacts—devices that “maintain, display or operate upon information” in order to, for example, assist us in cognitive tasks. The system view involves us seeing the actor, the task, and the artefact as a whole, whereas the system’s capacity is affected by the introduction of an artefact. From the personal view, however, which is the view of the human actor, their capacity is not necessarily enhanced by the artefact even if the capacity of the system is increased. As the task itself is changed, this can be experienced both positively and negatively by an actor, irrespective of the effect on the system in which they are a constituent part [16]. If we take this approach to the introduction of artefacts in general, we see that the different perspectives provide different perspectives on issues of automation and the introduction of AI in industry. From one perspective, humans are empowered, but from the other, the tasks are changed, and the actor may even feel diminished. Of importance is also the possibility that the capacity of AI may be said to have gone beyond the role of the cognitive artefacts here discussed. AI systems are often less of a help or tool for human actors, but more of an autonomous replacement.

Activity theory is another approach to HCI, in which both of Norman’s views are considered personal [15]. In activity theory, tools are seen to empower, and even change, the actor, and we focus in particular on the notion of mediation, internalisation, and externalisation of skills, as described in the literature on activity theory [49,50]. Kaptelinin [15] refers to studies that show that we often go through three phases when tools are used to assist us in tasks. First, we cannot effectively use to tool, so performance of the task is the same with or without the tool. In the second phase, we perform better with the tool than without. The third phase is the most interesting, and that is when we can perform the original task better than before, even without the tool [15]. Using the tools can actually change us and help us learn how to do new things. One example of how cognitive artefacts might help internalise new skills is how go and chess players are adapting their strategies and are now achieving new levels of skill by using computer software, such as AlphaGo and AlphaZero, in order to analyse, practice, and play in new ways [51,52,53]. In theory, AI systems with superhuman diagnostic abilities, mapping, and planning abilities for interventions and surgeries, VR goggles for training, augmented reality glasses used while operating in normal contexts, and also RAS systems might have similar effects, indicating that such systems do not make humans obsolete, but instead provide new avenues for human development.

While tools can empower individuals, technology also inevitably changes power relations, and structural power refers to the distribution of power in a given setting [54]. All technological change potentially impacts existing power structures, and the digitalisation in any sector inevitably involves shifts in power that must be examined when the effects of technology are discussed. An example of such effects of technology was seen when snowmobiles were introduced into Skolt Lapland, completely changing the economy of reindeer herding. The changes went far beyond the simple act of herding and gathering the reindeer, as it affected just about every aspect of Skolt societal institutions, social relations, economy, and the distribution of wealth and work [55]. Infrastructural changes such as these are most clearly linked to shifts of power, but also substitutional and more subtle changes involve shifts of power. While we mainly focus on the effects of new technologies on the technological infrastructure and individuals’ experience of work, we will continually also keep an eye on how the changes discussed changes power relations, as these are central to understanding the future of work. For example, automation in healthcare likely entails a power shift in favour of manufacturers and developers of digital technologies, a development that necessitates both political awareness and most likely various regulatory responses aimed at alleviating the potential negative consequences for both individuals and society of such power shifts. 

## 4. Discussion

With this framework in place, we will discuss how the examples and cases discussed in Section 2 relate to and entail changes at the three levels.

### 4.1. Skill and Task Transformation: The Micro Level

On the individual and organisational level, new technologies will inevitably lead to a transformation of the tasks and skills required to perform these. For example, RAS is similar to regular surgery, but it is certainly also different, and it requires a transition from old to new forms of surgery. Rather than manually performing surgery, the surgeon operates a machine, and, as a result, there is no longer a direct connection between the surgeon and the patient. The complex interplay between increasingly autonomous surgical robots and human surgeons transforms the tasks traditionally performed by the surgeon. As a result, the skills required from the human surgeon to ensure successful procedures will inevitably change [13,44]. When this leads to the need for fundamental educational change or the demand for entirely new types of workers, this impacts the macro level, as we return to below. However, some transformations are relatively minor, and workers and individuals continually adapt to the new forms of performing their jobs. For example, the introduction of robots in the eldercare facility might require limited training for the staff, but in most instances existing staff might be able to adapt and change to the new situations by, for example, improving their digital competence [56].

While RAS is rapidly evolving, there is no agreement on a specific learning curve, leading to disparities in the stakeholder’s training frameworks [57]. Subsequently, it is uncertain what certification is required to be considered a good robotic surgeon. Doctors and practitioners are exposed to using necessary medical equipment during their studies. However, the use of surgical robots is a relatively recent development. As a result, surgeons and practitioners must acquire the necessary skills to use surgical robots through external training. Basic training modules generally comprise patient side training, including correct patient positioning, port, sensor placement, robot docking, and console side training, including lab simulation and supervised operation control [58]. Presently, there are no standardised training modules for the use of surgical robots [59]. It implies that practitioners’ dexterity using surgical robots depends on the quality of the robot manufacturer’s training and may vary from one region to another. Researchers employing theories relating to the activity theory and the cognitive approach have proposed methods to enhance RAS training, and the focus in particular on the use of video combined with joint reflection as a beneficial approach [60]. The question here is whether, and to what extent, the insertion of robots in operating rooms will change the educational infrastructure surrounding medicine and clinical practice. Training and schooling changes as we move from low- to high-tech methods and technologies, but there is as of yet little to indicate that those capable of performing traditional surgery, for example, will not be able to learn to be effective are RAS.

### 4.2. Changes within Organisations and in the Quality of Care: The Meso Level

**Figure 1:**
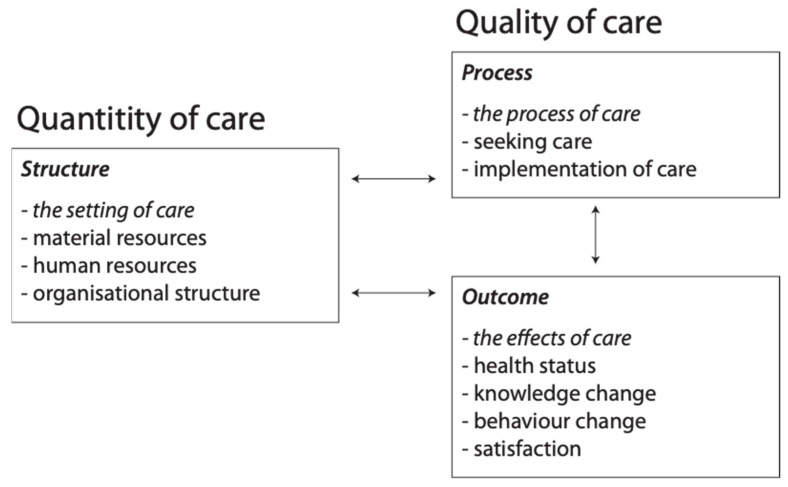
Quantity and quality of care [62].

There are several different areas in which digitalisation changes work in the healthcare sector. One important factor is whether new technologies are introduced in order to save costs, improve the quality of care, or both. One framework for analysing quality of care consists of three categories: structure, process, and outcome [61]. Resources are here considered as a part of structure, but in order to better illustrate the distinction between quality and quantity of care, Sætra [62] proposed rearranging the factors as shown in Figure 1:

As robots and other forms of automation are introduced in order to save costs, this might (a) reduce quality, (b) have no effect on quality, or (c) improve quality. Either way, in order to reduce costs through automation of tasks, humans must necessarily be made more effective—according to the system perspective of Norman [16]—or replaced by the new technology. If we use the example of nursing staff at an eldercare facility for patients with dementia, a social robot might be introduced to allow the caretakers to care for more patients, if the robot is effective in keeping the elderly calm and satisfied, and perhaps even provide them with certain forms of therapy or even physical assistance [62]. In such a situation, we could argue that workers are, from the system perspective, made more effective. One caretaker can care for a larger number of people when the system is perceived as consisting of the human being and the technological tools they use. However, from the personal view [16], the worker might not feel empowered or more effective, as the task of caring for the elderly has drastically changed with the introduction of new tools. In this sense, it is important to distinguish between the micro and meso levels. If the robot primarily performs repetitive tasks that are physically demanding but psychologically uninteresting, the worker might experience the tools as both liberating and positive, as this might allow their experience of work to improve as they will not have to perform heavy lifting, for example, and they might ideally free up some time for the more rewarding and stimulating parts of the job, such as interacting socially with the patients and providing them with therapy and social interaction. However, if the social robot takes over these parts of the job, the worker might experience the introduction of new tools as unfortunate, if this entails that time previously spent on rewarding tasks must now be spent on more tedious administrative tasks. In such a situation, automation is detrimental to how work is experienced, rather than automation leading to some form of dystopia [3].

If we examine the introduction of RAS from the perspective of a surgeon, we might conclude that this technology is empowering the surgeon while reducing the impacts invasive procedures have on the patients. A specialist surgeon will be able to operate far more effectively with this technology and they will even be able to operate remotely in the near future if technology continues to progress. If this is the case, it will free up time previously spent traveling and preparing. However, this change also clearly shows how new technologies entail shifts of power and distributional effects. While some surgeons will be empowered by RAS, a range of other surgeons will not be as well off as a result of this technology. While in hospitals top senior surgeons are highly valued, with the insertion of RAS, more junior surgeons with a shorter learning curve may master RAS sooner than more experienced doctors. However, with no dedicated training to it, current surgeons finishing their residence are getting the necessary skills through what has been called ‘shadow learning,’ e.g., YouTube videos and other informal training [63]. 

Moreover, the quantity of surgeries may increase as a result of this technological development, but the demand for surgeons overall might in fact decrease and become more specialised and specific. Surgeons will not be replaced by robots, per se, but the increased efficiency of RAS means that fewer surgeons will be able to perform the work previously carried out by a larger number of doctors, as also indicated by the high cost of the robotic systems themselves which entail that a large number of robot-assisted surgeries must be performed per year in order to make the purchase of RAS economically viable [64].

When automation leads to more effective use of resources, such technological change can be either substitutional or infrastructural [13]. In the context of healthcare, however, resource efficiency and increased productivity may not be the right parameters by which the technological uptake is evaluated, as these advancements also have broader and diffuse societal implications. How might the introduction of social robots and robots that assist caretakers in lifting patients, for example, affect the wider infrastructural conditions? The introduction of such technologies has clear and important effects *beyond* the changes that occur at the meso- and microlevel, as just discussed. Unless there are changes that relate to, for example, improvements in the quality-of-care to such a degree that it changes a society’s demographics (more people survive for longer and require care for longer), or the need for labour and/or type of competencies in the healthcare sector, the changes might merely be *substitutional.* While such changes can certainly be of great importance for individual workers and those that are cared for, and might have limited organisational implications, they might not affect the macro level in a significant way, at least for the moment.

The examples discussed indicate that most of the effects of digitalisation in the healthcare sector are limited to impact on the micro and meso levels. If this is indeed true, Barley’s [13] argument that classifying AI, big data, and modern robotics as drivers of the 4IE may have to be revisited. Social robots may, to a certain degree, substitute human caretakers [38,65], but as of yet there is no indication that they will eliminate the need for qualified human professionals in any area of the healthcare sector. On the contrary, the change is on another level, in the way how care is going to be delivered, whether medical doctors will spend more or less time with their patients, and whether the care administered is safer than before. With regards to artificial intelligence and diagnosis, we have seen that prophecies regarding the coming human obsolescence seem overstated [66], and it seems more likely that human–computer constellations will be the solution in most areas, and that these constellations involve making humans more effective while quality and reliability is increased. The same applies to RAS where it is the robot *assistance* that has proven to be effective, and not fully automated robotic surgery that eliminates the need for human specialists. 

### 4.3. Job Transformation: The Macro Level

#### 4.3.1. Task Replacement and Modification

Finally, we turn to the macro-level changes. A large quantitative study on industrial robots and human replacement shows a tendency towards worker replacement in industrial environments due to robots’ productivity effect [7]. Such developments are likely to apply to the healthcare sector as well, although so far, there has been no comprehensive data collected on the impact of robot use on healthcare worker replacement, most likely due to the relatively early stages of robot use in healthcare settings [48]. In 2015, the BBC released a software tool based on Frey and Osborne [8], which shows various occupations’ susceptibility to automation. Although this software was only based on one study, it gives some indication regarding the probability of automation of several healthcare-related jobs.

According to Frey and Osborne [8], medical practitioners and physiotherapists are said to run only a 2 or 2.1% automation risk, while other professions, such as dental nurses, nursery nurses or assistants, auxiliary nursing assistants, care workers, and home caregivers, have a much higher probability of replacement (60%, 55.7%, 46.8%, and 39.9%, respectively) compared to other jobs. Medical radiographers and dental technicians, for instance, are thought to be at a of 27.5% risk of being automated, ophthalmic opticians 13.7%, whereas paramedics or speech and language therapists are given lower risk ratings at 4.9% or 0.5%, respectively [8]. Other related professions also face a high risk of being automated. For instance, software healthcare practice managers’ and medical secretaries’ risks of automation are 85.1%, and hospital porters 57.3%. However, at this point, it is difficult to predict precisely what occupations and specific tasks are most likely to disappear or be replaced by machines and how this will translate into jobs that will be lost. Hence, Frey, and Osborne [8] suggest that any such figures should be taken with caution.

If jobs are lost, and not merely changed, such changes would likely lead to macro-level effects due to changes in skill requirements and employment patterns. However, while studies show that a large number of jobs are at risk of automation, the evidence as of yet seems to suggest that most predictions, regarding which jobs are theoretically susceptible to automation, seem to overstate this possibility. As with the case of radiographers—which were predicted to be fully replaced by machines by now—this has not come true [66], and rather than replacement, we have seen job transformations and the development of new constellations of human–machine interaction systems.

#### 4.3.2. Education and New Skill Requirements

While we have argued that changes on the macro level are somewhat limited at this point in time, the changes that occur on the meso and micro level do require certain changes in the education and organisation of the healthcare workforce already, which is part of the broader macro level. If these changes become substantial enough, they will lead to macro level impacts. 

One major effect of digitalisation and automation is that more workers will be required to have a basic or high level of digital skills. A surgeon doing RAS needs to understand how the machines they operate function, and this changes the requirement for surgeons rather drastically, as they have traditionally been required to have skills related mainly to anatomy and physically performing surgery. In addition, the caretaker that has traditionally cared for the elderly, provided them with food, social contact, and therapy, will need to be able to understand, supervise, and operate robots that perform these functions in order to be effective in this new situation. One key issue that was mentioned above is the growing importance of digital competence. One framework for understanding the various aspects of digital competence is the European Commission’s DigComp framework, which has been through a number of revisions and applied to different areas, such as school, work, and general citizens in general [67]. The five competence areas defined in this framework are: information and data literacy, communication and collaboration, digital content creation, safety, and problem solving. Of particular importance in relation to the developments described in this area are skills related to digital safety and problem solving, but also digital content creation, which includes skills related to programming, for example.

Another change implied by our examples is that human beings might, to a certain degree, be required to be good supervisors, administrators, and operators of machinery, rather than being specialists at the various skills they previously performed. This will certainly lead to certain new demands from various educations, as medical professionals will have to be increasingly proficient with technology, and technology developers may be required to know more about medicine. In an extreme form, we can imagine a situation in which certain educations become obsolete and various groups of occupations receive a unified and somewhat streamlined education that focuses on basic digital technology and administration. Since it can be anticipated that these technologies will have impacts at the macro-level, both professions can reasonably be expected to demand to be involved in, for example, political debates concerning the regulation of technology.

## 5. Discussion: Long Term Changes and Areas of Future Research

Digitalisation and automation are posed to make the healthcare sector more effective in terms of resources (both material and human), which might be used to improve the resource allocation in healthcare. For now, it seems that the near-future healthcare sector will be clearly recognisable and not radically different in organisation from what we see today, even if it will be more technical. We expect that the need for human beings in the sector will be relatively stable, but that the people who work in the sector in the future will have a different type of education, and that their responsibilities will often relate to translating and controlling the operation of advanced AI and robotic systems rather than on direct interaction with patients. Such a change may create an incentive structure that promotes larger institutions and efforts to garner the benefits of economies of scale, as these systems are (a) costly, and (b) have the potential to care for a large number of patients. 

As we have concluded that the changes at the macro level are as of now somewhat limited, we have also shown that there is a clear potential for important macro-level impacts in the future. Automation leading to job replacement and changed demands for education and competencies are two areas we have discussed here, with a particular emphasis on digital competence [67]. We also wish to point to three other areas of potential macro-level change that require more research.

First, there is a possibility that technological change leads to a change in how care is both perceived and delivered. While some have previously conceived care as something restricted to human–human relations, this might change, and our accompanying ideas of what constitutes *quality* care (refer to Figure 1 above) could simultaneously change [62]. Danaher [68] provides the foundation of such research in a recent article examining axiological futurism, in which value change as a result of technological change. In a similar vein, Sætra [69] deals with how robots designed for the use for love might change the very concept of love, and empirical research to test the strength of such hypotheses are required to accurately evaluate the fears related to certain negative effects of digitalisation in the healthcare sector. 

Secondly, while more digitalisation and automation allow for care to be delivered in new, and potentially more effective, ways, new challenges also manifest themselves. The need for digital skills will be important for workers, but technology is also susceptible to cyber-attacks which may have fatal consequences for, for example, patient safety [70]. Politicians, institutions, organisations, and individuals will all be required to account for such attacks. We might here also note that issues of privacy are highly relevant in this context, with the growth of sensor technology both in networked devices and surveillance equipment but also robots in general, which have a number of sensors and methods for storing and transmitting data [39]. Digital competence related to the protection of privacy, and more in-depth knowledge of how “digital dossiers” affect individuals facing these new technologies [71], are crucial for achieving a responsible and beneficial digitalisation process. In addition, there is always the risk of malfunction, and we as societies must decide what sort of systems of backup, for example, we demand, as old ways of performing tasks might relatively quickly be forgotten or become impractical once digitalisation and automation is implemented. If, or when, technology then fails, we must either accept such failure or require the potential for doing things the traditional way as well.

Thirdly, new technologies often require new interpretations of existing policies or the creation of new policy mechanisms to frame the developments accordingly. The EU, for example, recently proposed the AI Act as a regulation to align AI development with EU fundamental rights. All the developments in healthcare discussed in this article introduce new demands for legislators and policymakers. Digitalisation and automation are not some nature-given phenomena that simply occur. Or, rather, they do not have to be. We, as a society, have an opportunity to control and direct all the changes discussed, but this requires politicians to work proactively to understand the implications of new technologies and work actively with the industries and the healthcare sector in order to make sure that the future develops in a direction we desire. As argued by Sætra and Fosch-Villaronga [72], this should not entail preventing foundational research on AI or robotics, but instead actively regulating and legislating the application of such technologies. As shown in the various examples discussed in this article, digitalisation is associated with a number of important benefits for both patients, workers, and society in general, and it is imperative that the domains of science, ethics, and politics interact in such a way, allowing for these benefits to remedy the key challenges also created by the same technologies [72].

## 6. Conclusions

Technology is shaping the healthcare sector and changing it in a variety of ways. We have argued that, while AI, big data, and modern robotics are changing the healthcare sector, these changes are evolutionary and partly a logical consequence of techno-solutionism. A metaphor for understanding current technological development is travelling to some mountain by car; while the mountain in the distance seems to barely move, the markings on the road are advancing and passing at high speeds. This disconnect between the rapid advances and seemingly radical changes on the micro level (the markings on the road in this metaphor) and the delayed impacts on the macro level (the mountain) oftentimes amount to a disproportionate response that does not match the actual need created by these changes when they are properly analysed. Still, and inevitably, the insertion of technologies in any sector is not straightforward and has consequences for society in multiple levels that need an adequate response. 

This article has shown that, by applying a framework where effects on the micro, meso, and macro levels are distinguished from each other, the nature of technological change in the healthcare sector can be more clearly understood. Our approach allows for distinguishing between system and personal perspectives when examining effects on the micro level, which further helps understanding why change that may appear both radical and fundamental at the micro and meso levels are not necessarily associated with revolutionary macro-level changes. A key contribution of this article has thus been to show that a broad analytical perspective is required for understanding technological change and informing policymaking and society. Our work has also provided the foundation for further research of a more focused, and also empirical, nature. 

On the micro and meso level, individuals and organisations are experiencing changes, yet these changes do not, for the most part, involve the substitution of humans for machines, but rather a transformation of the skills and jobs that humans perform. As new human–machine partnerships are formed, workers are as of yet largely part able to keep up with these changes, which is why we argue that the changes to the macro level are somewhat limited. However, we show that the long-term effects of digitalisation do entail new requirements for digital competence, and these changes will, in the long term, have the potential to change the entire structure of the educational system, as the healthcare sector and other sectors increasingly require workers with medium-to-high-level digital skills and more administrative training. 

While our analysis undermines the assumption that digital technologies, and AI and robotics, in particular, constitute a fourth industrial revolution, their effects on the micro and meso level still require both political awareness and proper regulatory responses. By analysing technological transformation through the lenses of a layered approach, a better informed and proportionate response that calibrates societal expectations, preserves the rule of law, and avoids “regulatory madness” can be provided to guide society and technological development.

## Data Availability

Not applicable.
